# Teacher Cognition and Practice of Educational Equity in English as a Foreign Language Teaching

**DOI:** 10.3389/fpsyg.2022.820042

**Published:** 2022-03-03

**Authors:** Feifei Chen, Rohaya Binti Abdullah

**Affiliations:** ^1^Department of College English, Zhejiang Yuexiu University, Shaoxing, China; ^2^School of Educational Studies, Universiti Sains Malaysia, Penang, Malaysia

**Keywords:** English as a foreign language teaching, educational equity, teacher cognition, teacher practice, interrelations, factors

## Abstract

Teachers involved in English as a foreign language (EFL) teaching play a significant role in the process of moving toward educational equity. Teacher cognition is very influential in shaping teacher practice and thus affects students’ academic performance. However, although the role of EFL teachers as equity agents has been recognized, few studies have explored EFL teachers in-depth in terms of their cognition and practice. Moreover, no review studies have given sufficient attention to the task of elucidating the interrelations between EFL teachers’ cognition and practices in the context of educational equity. Therefore, to provide a novel perspective and generate fresh insights into this research field, the current study attempts to explain the connotations of these constructs, highlights the interplay between EFL teachers’ equity-oriented cognition and practice, and identifies both experiential and contextual factors that might have an effect throughout the teaching process. Finally, practical implications and directions for teachers, researchers, policy-makers, and social justice leaders who are interested in actualizing education as a means of attaining equity are also discussed.

## Introduction

Educational equity, a mandate from Sustainable Development Goal 4 (SDG 4), has long been considered to be an “important factor in enrichment of quality in education” (Borazjani and Bagheri, 2016, p. 61). Learning English as a foreign language (EFL) is established as a compulsory duty in many non-English-speaking countries and is viewed as an essential skill for global competence (Villegas, 2017). Therefore, under the backdrops of moving toward equitable education, EFL teaching is closely related to educational equity and exerts a significant influence on the process of minimizing educational and social gaps around the globe. Equity in EFL teaching can be promoted by ensuring equal opportunities for every learner (Lachance et al., 2019) and responding to the educational needs of diverse student populations (Leiva et al., 2021). However, inequities in EFL teaching persist and primarily manifest in teachers’ domination over the class without leaving students equal opportunities to become engaged in the teaching process (Chen and Vibulphol, 2019; Wu, 2019) and in the failure to understand learners’ needs by merely adopting a one-size-fits-all approach regardless of students’ identity markers (Du and Guan, 2016; Khdar et al., 2019), which may lead to low self-efficacy among students and discouraging learning outcomes (Mo, 2020).

Considerable attention has been given to equity issues occurring in the EFL teaching context; however, the majority of relevant research has been conducted from a macro perspective employing quantitative methods, debating the educational inequities triggered by factors such as disadvantaged learners’ gender (Meshkat and Nejati, 2017; Ariyanto, 2018; Brutt-Griffler and Kim, 2018), race (Von Esch et al., 2020; Xiang and Yenika-Agbaw, 2021), and socioeconomic status (Lee, 2020; Lorenzo et al., 2020; Yao, 2021). In contrast, limited attention has been given to the role of a subtle yet critical link—EFL teachers, who play a pivotal role in maintaining a fair and inclusive education for all learners.

The interplay between EFL teacher cognition and teacher practice has been noted (Wang and Ryan, 2020). Research has supported the claim that EFL teachers’ cognition can be very influential in shaping their instructional practices, thereby affecting the learning outcomes of students (Kang and Cheng, 2014; Mohammadi and Moradi, 2017; Gao et al., 2020). Accordingly, teachers’ equitable practices under the guidance of their equity-oriented cognition can keep learners “intellectually, socially, and psychologically engaged” (Schreiner, 2014, p.10). Given the importance that teacher cognition and practice have for creating an equitable and harmonious learning atmosphere, teachers who undertake the task of EFL teaching should act as equity agents to help each individual achieve his or her full potential (Guo et al., 2019).

Despite the fundamental role that teachers play in promoting educational equity in EFL teaching, only a few studies have investigated the association between teacher cognition and practice from an equity perspective. Furthermore, hitherto, there have been no review studies aimed at investigating these constructs and shedding light on the inner association between them. As Fullan (2001) argued, the success of educational reforms depends on teachers’ thoughts and actions. In line with this claim, the present study seeks to address this gap by constructing transformative visions for educational equity and elucidating the interrelationships between teacher cognition and practice that are pertinent to educational equity in EFL settings. More specifically, the study presents research findings that identify the factors influencing teachers’ equity-oriented cognition and practice, which may help researchers develop investigational instruments to conduct in-depth studies.

## Definition of Educational Equity

**Figure 1:**
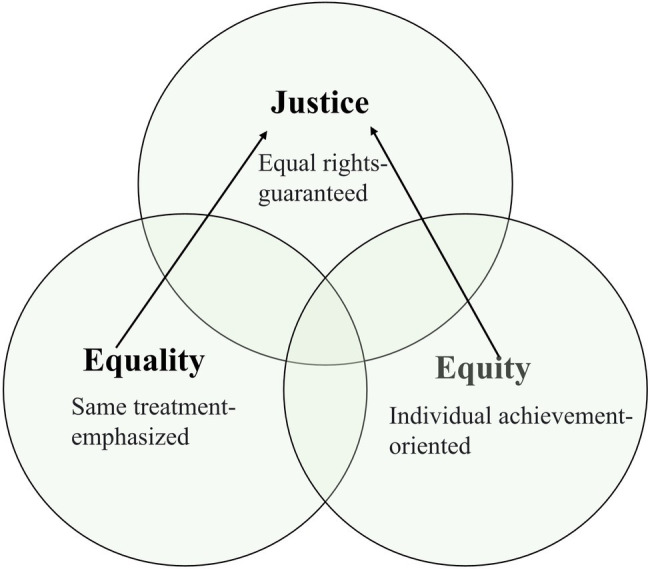
Boolean diagram of the overlapping terminology.

The concept of educational equity has attracted much attention in recent years (Scheurich et al., 2017). To attain a correct understanding of this notion, the keyword “equity” should first be clarified. However, in some sense, the word “equity” is often interpreted in a confusing and problematic way, being used interchangeably with the words “equality” and “justice” (Panthi et al., 2018; Rasooli et al., 2019; Shaeffer, 2019), and these latter two terms are also closely related (Robeyns, 2009). These concepts, although they overlap in certain dimensions, are distinct in the context of educational goals and purposes. Specifically, “equity” focuses more on obtaining what the learner needs to achieve success (Minow, 2021), indicating objective assessments and subjective morals, while “equality” mainly refers to receiving the same treatment and resources and has normative overtones (Smith et al., 2017). Both “equity” and “equality” aim to ensure “justice,” which emphasizes the “demands for equal rights under the law” (Stoll, 2011, p. 36). To illustrate this overlapping terminology, a Boolean diagram is provided (Figure 1).

Inspired by Coleman et al. (1966), who proposed establishing equality of educational opportunities to address equity issues, several researchers, such as Gipps and Stobart (2009), Klenowski (2009), and McLaughlin (2010), have made similar claims that a situation in which equal educational opportunity is granted to everyone constitutes the essence of “educational equity.” Similarly, Blankstein et al. (2016, p. 3) defined that term as a “commitment to ensure that every student receives what he or she needs to succeed academically.” There is consensus among scholars that the term educational equity, which has richer implications (Barrow and Grant, 2019), depicts a situation in which each individual, irrespective of race, gender, family background, or any other personal marker, can be endowed with an equal opportunity to fulfill his or her academic potential (McLaughlin, 2010).

Based on a widely accepted explanation, the term contains two levels of meanings, “fairness” with respect to “achieving the educational potential” and “inclusion” with regard to “ensuring a basic minimum standard of education for all” (OECD, 2008, p. 2). As such, educational equity serves as both a means and an end, a principle and an ideal, a process and an outcome, and it requires teachers, educators, and policy-makers to make joint efforts to minimize students’ academic gaps to allow them to realize their potential. As Campbell (2020, p. 17) noted, narrowly defining equity in terms of outcome performance may keep people from truly understanding “the wider contexts, processes and outcomes of inequity.” Therefore, a proper interpretation of educational equity that outlines the need for an equal chance at academic success can help solve the conceptual dilemma and thus further promote justice within the educational context.

## Teacher Cognition Regarding Equity

Teacher cognition, which is initially shaped by a teacher’s schooling and professional experiences, refers to cognitive constructs such as knowledge, beliefs, and thoughts (Borg, 2003, 2013). Considering the tremendous impact of teacher cognition on practices, some researchers have attempted to clarify the nature of teachers’ equity-oriented cognition (Dweck, 2010; Ramaley, 2014; Schreiner, 2014; Brinegar et al., 2018; Skerrett et al., 2018; Nadelson et al., 2019; Stembridge, 2019). Dweck (2010) stated that teachers who are focused on equity should not only claim to have faith in their students’ abilities to learn but also show a commitment to the task of finding a way to facilitate such learning. Inspired by Dweck, Ramaley (2014) highlighted the importance of equity-oriented cognition for teachers to reflect on what is needed to help students feel a sense of belonging and on other factors that can help meet the linguistic and sociocultural needs of students from different backgrounds. His view is expressed by the statement that equity-focused teachers expect to “empower each student to transform themselves” (Brinegar et al., 2018, p. 2). However, these studies failed to explicitly describe the nature of equity-oriented cognition.

Later, Nadelson et al. (2019) further elaborated on the concept by using quantitative and qualitative approaches. Apart from student-centered learning, informal leadership, advocacy for equity needs, and culturally responsive teaching were also identified as integral components of teachers’ equity mindsets. In accordance with the arguments made by Gay (2000), teachers should be culturally conscious and sustaining to create an impartial and inclusive learning atmosphere. The study was innovative in that it crystalized the six attributes of teachers’ equity cognition; however, its findings were limited to a small number of cases. As a result, a model of teachers’ equity-oriented cognition that can facilitate teaching must be validated and developed by further research.

The importance of equipping EFL teachers with equity-oriented cognition in a multicultural society cannot be overemphasized. As Lucas and Schecter (1992) noted, to teach toward equity, teachers must become aware of students’ sociocultural diversity and introspective regarding their own values, beliefs, and identities within the whole educational process, and then, they must incorporate these elements into their teaching. Indeed, recognizing the vital links between EFL teachers’ cognition and practices is a first step to preparing teachers for inclusive and effective teaching.

## Teacher Practice of Equity

Teacher practice, as noted by Woods (1996), is the application or employment of teachers’ knowledge, beliefs, and thoughts in classroom teaching. Under the global discourses of neoliberalism, an increasing number of studies aiming to illuminate English teachers’ equitable practices have emerged. Banks (1995, p. 152) defined equity pedagogy as teaching strategies and classroom environments that can help even disadvantaged and marginalized students to become “reflective and active citizens of a democratic society.” To that end, teachers should be interest-driven and student-centered, prioritizing students’ linguistic and sociocultural needs, offering them equal learning resources and opportunities, and advocating group communication and collaboration so that every student can be engaged in a fair and just learning environment (Gay, 2013; Januszyk et al., 2016). By revisioning and reenacting contemplative equity pedagogy from a humanity lens, Powietrzynska et al. (2021) advocated for building rational trust and authentic relationships between teachers and students to embrace equity.

To give an example of equitable pedagogical practice, Dyches and Sams (2018, p. 373), who perceived the context-bound “pedagogical idealism” of teachers as “an orientation to teaching that aspires to equity and justice for all students,” exemplified a realist approach that combined equitable teaching with traditional materials. In addition, certain equity frameworks, such as culturally relevant pedagogy (Ladson-Billings, 1995), culturally responsive teaching (Gay, 2000), and equity literacy (Gorski, 2017), can also provide practical strategies to sustain an inclusive and just learning atmosphere for culturally and linguistically diverse students in an era of standards-based and teacher-dominated teaching.

Likewise, the importance of promoting equitable teaching among EFL teachers is self-evident. Without explicit teaching methods to guide teachers’ practices toward fairness and inclusion, teachers will inevitably exhibit bias toward learners at different levels (Staats et al., 2016). Educational equity, as noted by Dyches and Boyd (2017, p. 12), should “pulsate throughout every instructional maneuver.” Therefore, to create a fair learning environment for all, ongoing studies are needed to construct a clear picture of equitable pedagogy among EFL teachers.

## Interrelations Among Educational Equity, EFL Teachers’ Cognition, and Practices

The increase in the number of English language learners worldwide has made (in) equity in the English teaching context a critical issue that must be properly addressed (Murray, 2020). Conceivably, the focus on research concerning educational equity has shifted from the macro-level of fair access to education for all to the micro-level that mandates that everyone receive what they need to fulfill their learning potential. Under such a transformative context, the active forces—teachers—stand at the heart of ensuring fairness and justice in the educational process (Crawford-Garrett, 2017). Thus, to build a more “democratic, participatory, equitable, professional, and egalitarian future” for English language teaching (Yazan and Rudolph, 2018, p. 6), EFL teachers should have correct cognitions regarding equity and should engage in relevant equitable pedagogy in actual classrooms.

EFL teachers’ instructional practices have been shown to be influenced by their implicit and intricate cognition (Borg, 2003; López-Barrios et al., 2021). When teachers provide instruction in the context of actual classroom teaching, apart from being influenced by contextual factors in and outside the classroom, they are also subject to their cognitive constructs, such as knowledge, beliefs, and thoughts. In Borg’s framework (Borg, 2003), three elements, namely, teacher cognition, teacher practice, and contextual factors, dynamically interact with each other throughout the teaching process.

**Figure 2:**
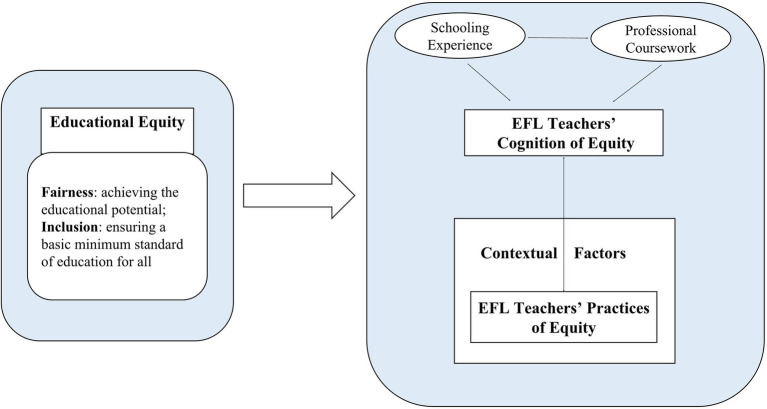
Interrelationship among educational equity, EFL teacher’s cognition, and practices (adapted from Borg, 2003; OECD, 2008).

Taken together, to provide education for all, EFL teachers should articulate the importance of equal opportunities for each learner and, in the meantime, show great commitment to the task of facilitating such opportunities (Guo et al., 2019). In simple terms, EFL teachers should be equipped with equity-conscious cognition as part of their mindsets when engaging in instructional practices. Only when equity gaps are borne in mind can teachers successfully ameliorate the educational inequities embedded in their instructions (Orfield, 2014). Based on the above discussion and Borg’s language teacher framework, a framework that can highlight the interrelations among the constructs of educational equity, EFL teachers’ cognition, and practices is proposed (Figure 2).

## Empirical Studies Concerning the Interrelation Between Equity-Related Teacher Cognition and Practice

Although teacher cognition and practices have been a recurring theme in the EFL context, only a few researchers have attempted to investigate the interrelations between teachers’ cognition and practice that are pertinent to educational equity. A small-scale case study by Posti-Ahokas and Janhonen-Abruquah (2021) reported that equity-centered practices could only occur if teachers were aware of the causes of inequities and the impact of inequities on teacher positionality. That is, teachers’ equity-bound cognition is a prerequisite that can guarantee equitable practices. In contrast to this statement, a growing body of studies (Chen and Vibulphol, 2019; Wei and Cao, 2020; Irgin, 2021; Wu et al., 2021) has found a distinctive mismatch between EFL teachers’ cognition and practices; this dissonance is also reflected in teachers’ cognitions and practices toward equity (Erling et al., 2021).

Notably, the mismatch in question here, which has become quite serious, lies in the fact that although teachers report that they have adopted a student-centered stance, they act in precisely the opposite way during their teaching. In an empirical qualitative study investigating four Chinese EFL teachers’ instructional decision making, the findings showed that although teachers intended to build an active and fair environment for all, their actual teaching was still teacher-centered rather than equity-oriented (Zhang and Yang, 2017). In a similar vein, the findings of one mixed study showed that although EFL teachers self-reported that they recognized equity as one of the most fundamental guidelines to achieve social justice when interacting with students in the classroom, they were subject to strong biases in terms of students’ genders, personalities, social backgrounds, and English-speaking abilities (Chen, 2020). Previous studies have revealed that although EFL teachers self-report being aware of equity, their practices are still far from satisfactory (Maeng and Lee, 2015).

Certain key experiential and contextual factors have been reported to account for the hidden mechanism that triggers the incongruence between EFL teachers’ equity-related cognition and practice. Drawing on the framework of Borg (2003), experiential factors are mainly associated with teachers’ learning and professional experiences that shape their initial cognition of teaching. Using a qualitative approach to explore two Indonesian EFL teachers’ cognition concerning the incorporation of equity into their teaching, Sulistyowardani et al. (2020) revealed that teachers’ own learning experiences and personal beliefs were major factors shaping teachers’ cognition regarding justice and thus affected their practices. The findings are consistent with those of Kaur (2012, p. 487), who maintained teachers’ former experiences, beliefs, and perspectives could “re-inscribe stereotypes and perpetuate the social and historical inequities.” Regarding contextual factors, evidence has suggested that curriculum demand can transform EFL teachers’ cognition toward equity (Sulistyowardani et al., 2020). Moreover, researchers (Wang and Gao, 2013; Grudnoff et al., 2017) identified that the reason teachers often fail to realize the importance of equity is the inadequacy of teacher education programs concerning equity and justice. Especially in China, EFL teachers who are deeply influenced by Confucianism (Sun and Zhang, 2021), the dominant philosophical paradigm that asserts teachers’ authority over students (Gao, 2020), and utilitarian exam-oriented doctrines by which students’ scores matter most (Liu et al., 2019; Mo, 2020) may somehow demonstrate a disjuncture between equity-ended cognitions and practices.

## Implications and Directions for Future Studies

To conclude, the main goal of the current study was to generate fresh insights for any parties concerned with the research field of EFL teachers’ cognition and practice regarding equity. The three constructs, namely, educational equity, EFL teachers’ cognition, and practices of equity, were explained in detail. Moreover, the intricate connection between EFL teachers’ cognition and practices pertinent to educational equity was also illuminated with the support of theoretical and empirical evidence. From the literature reviewed, it can be inferred that within an educational context characterized by inequities and injustices, there is often a mismatch between EFL teachers’ claimed cognitions and actual classroom practices regarding educational equity. Factors that cause such a mismatch can be both experiential (referring to teachers’ learning and professional experiences) and contextual (including curriculum demand, teacher education programs, teacher authority, and exam-oriented doctrines). However, facilitating factors seem to have been investigated only narrowly and deserve further exploration.

These findings can be thought provoking for EFL teachers who are involved in the global educational system to reflect on their pedagogical thoughts and deeds. Given the crucial role teachers play in dismantling inequities that hinder students’ development and in closing the academic gap, it is a good sign that equity, as a common concern in the English language teaching area (Erling et al., 2020), has become a long-term pursuit for EFL teachers with equitable visions both at home and abroad. Furthermore, since equity is perceived as an important predictor of students’ self-efficacy (Daemi et al., 2017), this study has important implications for educational experts and policy-makers concerning how to include more voices from culturally and linguistically diverse students and how to verify whether teachers are incorporating equity into their classroom practices (Riordan et al., 2019). As claimed by Andrews et al. (2017), inequity will be amplified in schools if poorly qualified teachers continue to teach the most disadvantaged students. Therefore, to make instructional equity a reality, researchers or social justice leaders who are interested in actualizing education as a means of promoting equity should continue to explore the facilitating factors that constitute English teachers’ equity-focused cognitions and practices by using qualitative methods in longitudinal studies.

## Author Contributions

FC read through the relevant literature and shed light on the definition and connection between teacher cognition and practices. RA provided insightful suggestions. All authors contributed to the article and approved the submitted version.

## Conflict of Interest

The authors declare that the research was conducted in the absence of any commercial or financial relationships that could be construed as a potential conflict of interest.

## Publisher’s Note

All claims expressed in this article are solely those of the authors and do not necessarily represent those of their affiliated organizations, or those of the publisher, the editors and the reviewers. Any product that may be evaluated in this article, or claim that may be made by its manufacturer, is not guaranteed or endorsed by the publisher.
